# Human cutaneous neurofibroma matrisome revealed by single-cell RNA sequencing

**DOI:** 10.1186/s40478-020-01103-4

**Published:** 2021-01-07

**Authors:** Jean-Philippe Brosseau, Adwait A. Sathe, Yong Wang, Toan Nguyen, Donald A. Glass, Chao Xing, Lu Q. Le

**Affiliations:** 1 Department of Dermatology, University of Texas Southwestern Medical Center At Dallas, Dallas, TX 75390-9069 USA; 2 Eugene McDermott Center for Human Growth and Development, University of Texas Southwestern Medical Center At Dallas, Dallas, TX 75390-9069 USA; 3 Department of Bioinformatics, University of Texas Southwestern Medical Center At Dallas, Dallas, TX 75390-9069 USA; 4 Department of Population and Data Sciences, University of Texas Southwestern Medical Center At Dallas, Dallas, TX 75390-9069 USA; 5 Simmons Comprehensive Cancer Center, University of Texas Southwestern Medical Center At Dallas, Dallas, TX 75390-9069 USA; 6 UTSW Comprehensive Neurofibromatosis Clinic, University of Texas Southwestern Medical Center At Dallas, Dallas, TX 75390-9069 USA; 7 Hamon Center for Regenerative Science and Medicine, University of Texas Southwestern Medical Center At Dallas, Dallas, TX 75390-9069 USA; 8 Present Address: Department of Biochemistry and Functional Genomic, Centre de Recherche du Centre Hospitalier de Universitaire de Sherbrooke, Université de Sherbrooke, Sherbrooke, Canada

## Abstract

Neurofibromatosis Type I (NF1) is a neurocutaneous genetic syndrome characterized by a wide spectrum of clinical presentations, including benign peripheral nerve sheath tumor called neurofibroma. These tumors originate from the Schwann cell lineage but other cell types as well as extracellular matrix (ECM) in the neurofibroma microenvironment constitute the majority of the tumor mass. In fact, collagen accounts for up to 50% of the neurofibroma’s dry weight. Although the presence of collagens in neurofibroma is indisputable, the exact repertoire of ECM genes and ECM-associated genes (i.e. the matrisome) and their functions are unknown. Here, transcriptome profiling by single-cell RNA sequencing reveals the matrisome of human cutaneous neurofibroma (cNF). We discovered that classic pro-fibrogenic collagen I myofibroblasts are rare in neurofibroma. In contrast, collagen VI, a pro-tumorigenic ECM, is abundant and mainly secreted by neurofibroma fibroblasts. This study also identified potential cell type-specific markers to further elucidate the biology of the cNF microenvironment.

## Introduction

Neurofibromatosis type I (NF1) is a neurocutaneous genetic disorder with a frequency of 1 in 3000 births. This disease is characterized by the development of skin lesions called cutaneous neurofibromas (cNFs) [1]. Neurofibroma develops as the result of biallelic inactivation in the *NF1* tumor suppressor gene in the Schwann cell lineage, leading to an increase in Ras signaling. Although cNF is a benign tumor with zero malignant potential, it is often disfiguring and a great source of anxiety for NF1 patients. Surgical removal is the only treatment available, but it is impractical in patients with hundreds or thousands of tumors covering their bodies. Thus, there is an urgent need to develop effective therapies to reduce tumor burden.

On the one hand, strategies aimed at targeting the upstream or downstream pathways of Ras signaling in Schwann cells have not been very effective at regressing cNF [2]. On the other hand, independent laboratories have demonstrated that the microenvironment modulates neurofibroma development, thus making it a potential target for treatment. Mice with a heterozygous mutation for *Nf1* (mimicking NF1 patients) develop neurofibroma faster than their wild type littermates [3–5]. The cellular and molecular mechanisms by which the microenvironment promotes neurofibroma development, however, is unclear [6]. The neurofibroma microenvironment is composed of fibroblasts, pericytes, immune cells (such as macrophages, mast cells), and blood vessels mingled in a thick collagenous matrix. Although mast cells have been reported to be potential key players [1, 4, 7, 8], the vast majority of NF1 patients did not respond to a mast cell inhibitor in a clinical trial [9]. In addition to these various cellular components, neurofibromas contain a dense extracellular matrix (ECM) deposit, especially collagens: pioneering work by Peltonen and co-workers reported that up to 50% of a neurofibroma's dry weight is collagen as judged by the amount of hydroxyproline found in neurofibroma [10]. Although they further confirmed the presence of collagen type III [11, 12], IV [11, 13], V [11], and VI [14, 15] by in situ hybridization and immunohistochemistry, these techniques are rather qualitative. Because fibroblasts are collagen type I producers by definition, it is assumed that the bulk of collagen in neurofibroma is of collagen type I. Since fibrosis is by definition an excess of collagen I deposit, one leading hypothesis is that neurofibroma is similar to a nerve fibrosis [1] or a nerve injury that never heals [16]. However, recent clinical trials using the anti-fibrotic pirfenidone to treat plexiform neurofibromas had very modest results [17]. It is unclear if pirfenidone was ineffective at reducing collagen I deposition or if collagen I is simply not required for neurofibroma maintenance. Therefore, while the microenvironment appears critical for neurofibromagenesis, the exact cell type(s) and factor(s) involved in ultimately signaling back to the tumorigenic *NF1*^−/−^ Schwann cells remain unknown.

A major limitation to understanding the role of the microenvironment in neurofibroma biology is the lack of markers to distinguish its cell types. Not surprisingly, assessing the cellular source of the neurofibroma matrisome in vitro has proven to be difficult [18]. However, with the advent of single cell transcriptome analysis (scRNA-Seq), it is now possible to unbiasedly determine the cell type composition of a tissue [19, 20]. Here, we applied scRNA-Seq technology to fresh human cutaneous neurofibroma (cNF) to evaluate the cellular and molecular composition of the microenvironment. Our analyses revealed the type of ECM secreted by each cell type, provided a complete profiling of cNF collagen, and identified specific cNF fibroblast markers that will provide a molecular platform to further explore the biology of cutaneous neurofibroma.

## Results

### Single cell analysis of human cutaneous neurofibroma

**Fig. 1:**
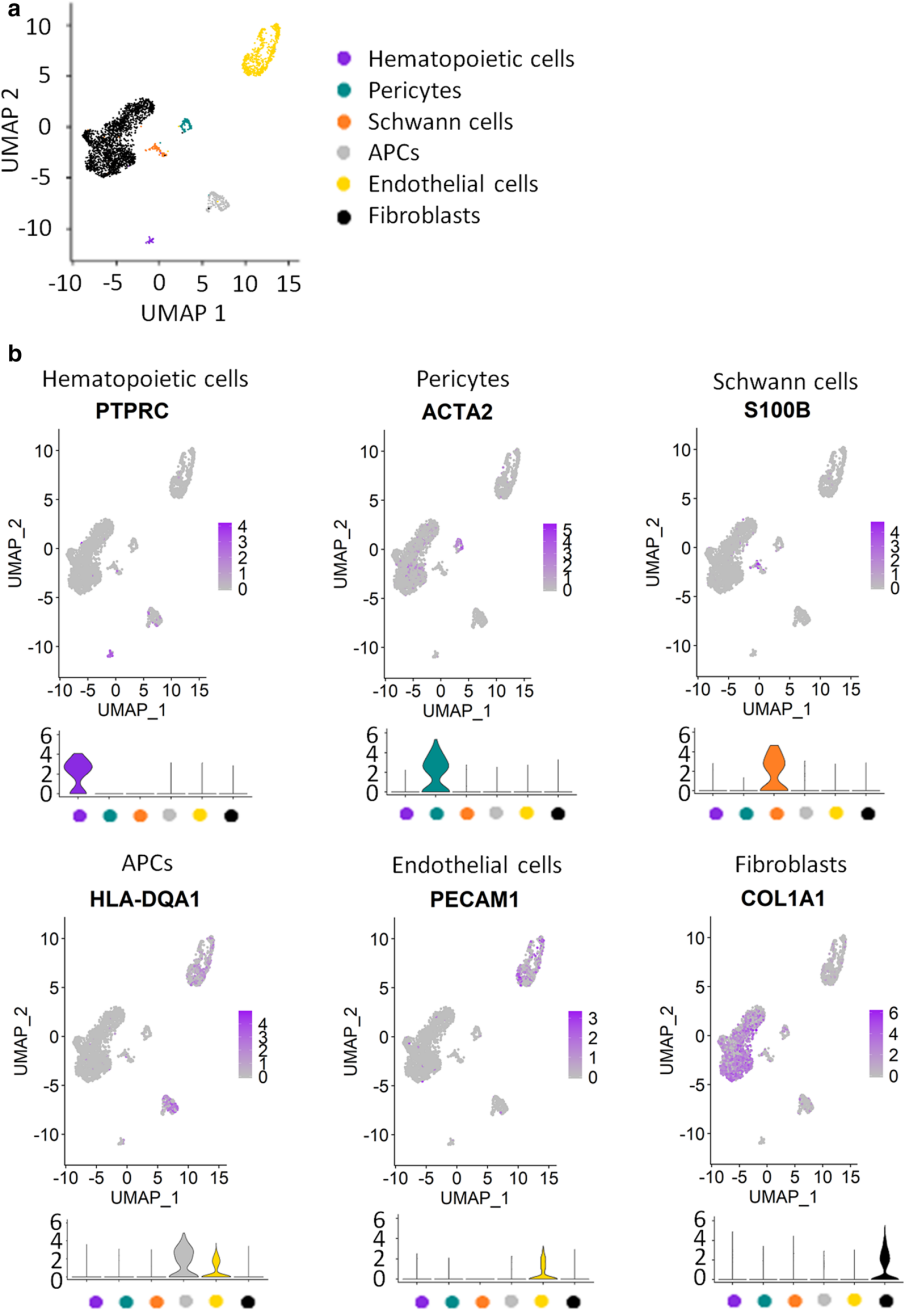
Single cell analysis of human cutaneous neurofibroma identifies 6 major cell types. **a** Uniform Manifold Approximation and Projection (UMAP) shows groupings of three human cutaneous neurofibroma cell populations totaling 3000 cells (random sampling of 1000 cells per sample). Each point represents a cell. Cells are color-coded according to cell type; 64 hematopoietic cells, 96 pericytes, 95 Schwann cells, 230 antigen-presenting cells (APCs), 619 endothelial cells, and 1896 fibroblasts were identified. **b** Feature plots (upper) and violin plots (lower) of genes defining different cell types in human cutaneous neurofibroma. The intensity of the purple color indicates the normalized level of gene expression

To determine the repertoire of ECM genes and ECM-associated proteins (i.e. the matrisome) [21] expressed in cNF, we performed scRNA-Seq. Initially, we optimized a protocol that allows cell extraction with high yield and viability coupled to the 10XGenomics technology for scRNA-Seq. This protocol was applied to fresh human cNFs at the globular stage and yielded a total of 17,132 transcriptomes (cells) across 3 samples. We analyzed each sample individually for quality control. To identify the shared clusters (and associated cell types) we integrated all the samples using Seurat. To ensure that every sample is homogeneously represented and analyzed, we randomly subsampled 1000 cells per sample, totaling 3000 cells (Additional file 1: Figure S1A). We also verified this analysis without subsampling and got the same results (Additional file 1: Figure S1B). We further carried out subclustering as described in the “Methods” section. The UMAP (Uniform Manifold Approximation and Projection) visualization of this clustering analysis is shown in Fig. 1a. We identified the markers per cluster (“Methods” section) and annotated the cell identity of the resulting clusters by manually surveying their global gene expression (Additional file 2: Table S1). As expected, we identified a cell cluster corresponding to the tumorigenic Schwann cells, i.e. positive for Schwann cell markers [*S100B, CDH19, PLP1*], as well as clusters from non-tumorigenic cells of the microenvironment: endothelial cells [*PECAM1* (CD31)*, CD74, CLDN5*], hematopoietic cells [*PTPRC* (CD45)*, CCL5, CD69*], pericytes [*ACTA2* (SMA)*, MCAM* (CD146), *RGS5*], antigen-presenting cells (APCs) [HLA-DRA, HLA-DQA1, HLA-DPB1] and fibroblasts [*COL1A1, DCN, LUM*] (Fig. 1b, Additional file 1: Figure S2 and Additional file 2: Table S1). Overall, our scRNA-Seq data confirmed the presence of all expected cell types within cNF.

### The cellular source of the neurofibroma matrisome

**Fig. 2:**
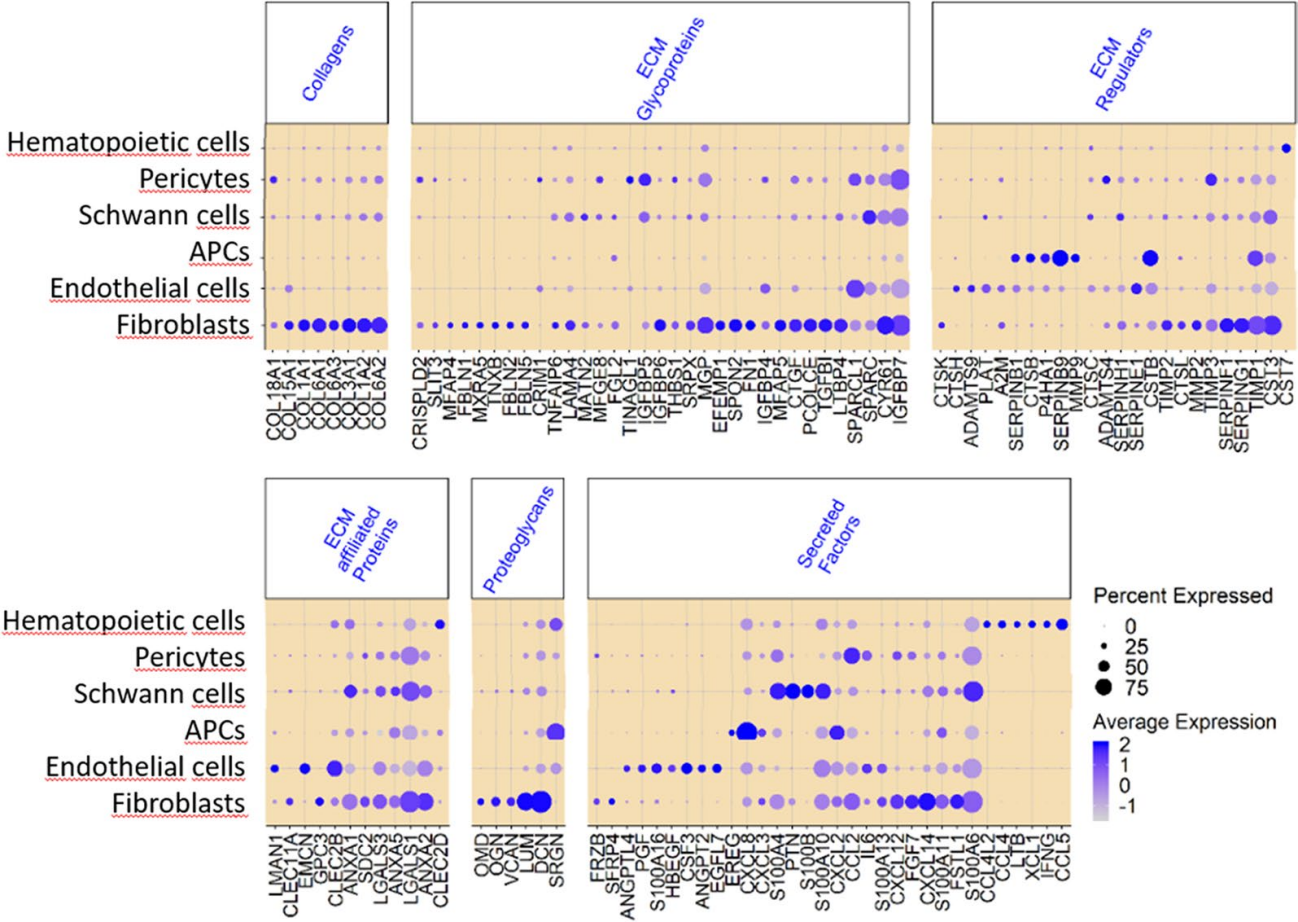
Cell types within the cNF microenvironment show overlapping and unique expression of matrisome genes. **a** Dot plot representing the shared and common matrisome genes expressed in neurofibroma hematopoietic cells, pericytes, Schwann cells, antigen-presenting cells (APCs), endothelial cells, and fibroblasts

Next, we investigated which cell types contribute to the neurofibroma matrisome. To do this, we retrieved the top markers for each of the six cell clusters (Additional file 2: Table S1) as mentioned earlier and filtered for gene expression signatures for any human matrisome genes (http://matrisomeproject.mit.edu/other-resources/human-matrisome/). This yielded a list of 115 matrisome genes, with some uniquely expressed and some shared across the six neurofibroma cell types (Fig. 2). With the goal of identifying putative cell type-specific markers, we examined the genes in Fig. 2. In addition to the general cell type markers presented in Fig. 1b, we found that hematopoietic cells specifically/uniquely express cystatin F (*CST7*); pericytes express the tubulointerstitial nephritis antigen like 1 (*TINAGL1*); endothelial cells express the EGF like domain multiple 7 (*EGFL7*); and fibroblasts express fibronectin 1 (*FN1*). Thus, all neurofibroma cell types contribute to the ECM deposition, and some matrisome genes are potential markers for identifying the cell types that populate the neurofibroma microenvironment.

### Classic fibrogenic fibroblasts are rare in neurofibroma

**Fig. 3:**
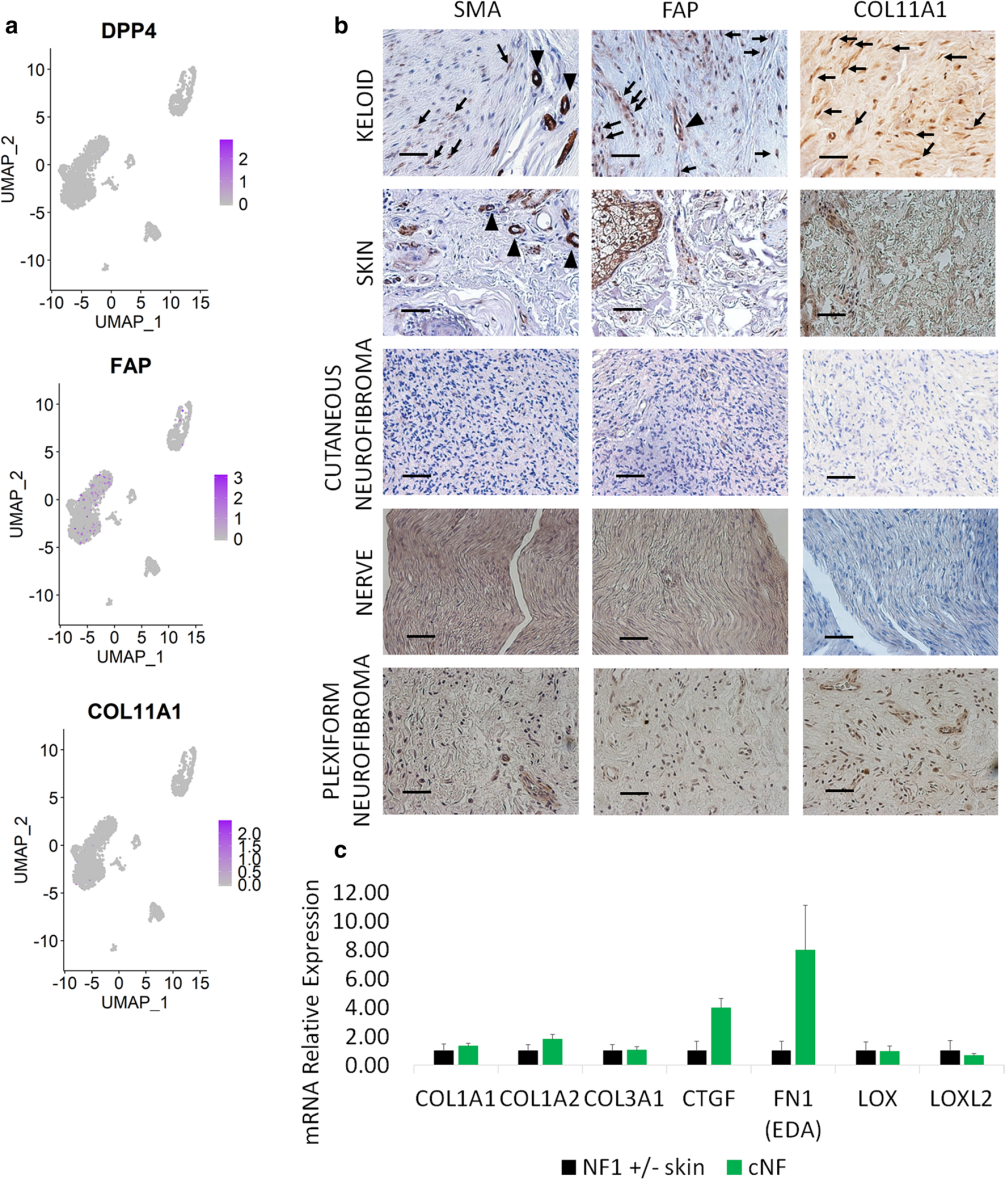
Classic fibrogenic fibroblasts are rare in neurofibroma. **a** Feature plots of dipeptidyl peptidase 4 (*DDP4*), fibroblast-activated protein (*FAP*), and *COL11A1* in human cutaneous neurofibroma. The intensity of the purple color indicates the normalized level of gene expression. **b** Characterization of activated fibroblasts in human clinical samples [keloids, normal skin (skin), cutaneous neurofibroma (cNF), normal peripheral nerve (nerve), and plexiform neurofibroma (pNF)] by immunohistochemistry [alpha smooth muscle actin (SMA), Fibroblast activation protein (FAP), and COL11A1]. **c** Profiling of the universal pro-fibrotic gene expression signature in human cutaneous neurofibroma (n = 5) and their normal skin margin (n = 5) by real-time PCR. Scale bar = 50 um. Arrows point fibroblasts and arrow heads point pericytes unspecific staining inherent to these fibroblasts markers

All major organ fibrosis and many cancer-associated fibroblasts are characterized by the expression of the smooth muscle actin (SMA) marker. SMA is also used as a pericyte marker [22] and our scRNA-Seq data confirmed this (Fig. 1b), while revealing that very few cNF fibroblasts are SMA positive. These data indicate that classic fibrogenic fibroblast markers are rare in cNF. Indeed, dipeptidyl peptidase 4 (*DPP4*), fibroblast activated protein (*FAP*), and collagen type XI (*COL11A1*), markers of activated/cancer-associated fibroblasts [23–25], are expressed at very low levels (Fig. 3a). These findings were validated in human tissues using keloid as a positive control. Keloid is also a benign skin tumor but characterized by SMA-positive fibroblasts and TGFβ activation [26, 27]. Importantly, the plexiform type of neurofibroma (pNF), which differs clinically from cNF [1], is also negative for these fibrogenic markers, generalizing the findings beyond the cutaneous type of neurofibroma (Fig. 3b). To further demonstrate the lack of classic fibrogenic fibroblasts and associated markers in neurofibroma, we profiled the universal fibrogenic expression signature [28] in bulk cNF (n = 5) and their matched normal skin margin (n = 5) by qPCR. As suggested by the scRNA-Seq analysis, the majority of the markers surveyed are not dramatically increased in cNF (Fig. 3c). Once again, these data indicate that the majority of cNF fibroblasts are different from the classic fibrogenic fibroblasts and hence, express a different set of markers.

### Neurofibroma fibroblasts do not abundantly secrete collagen type I

**Fig. 4:**
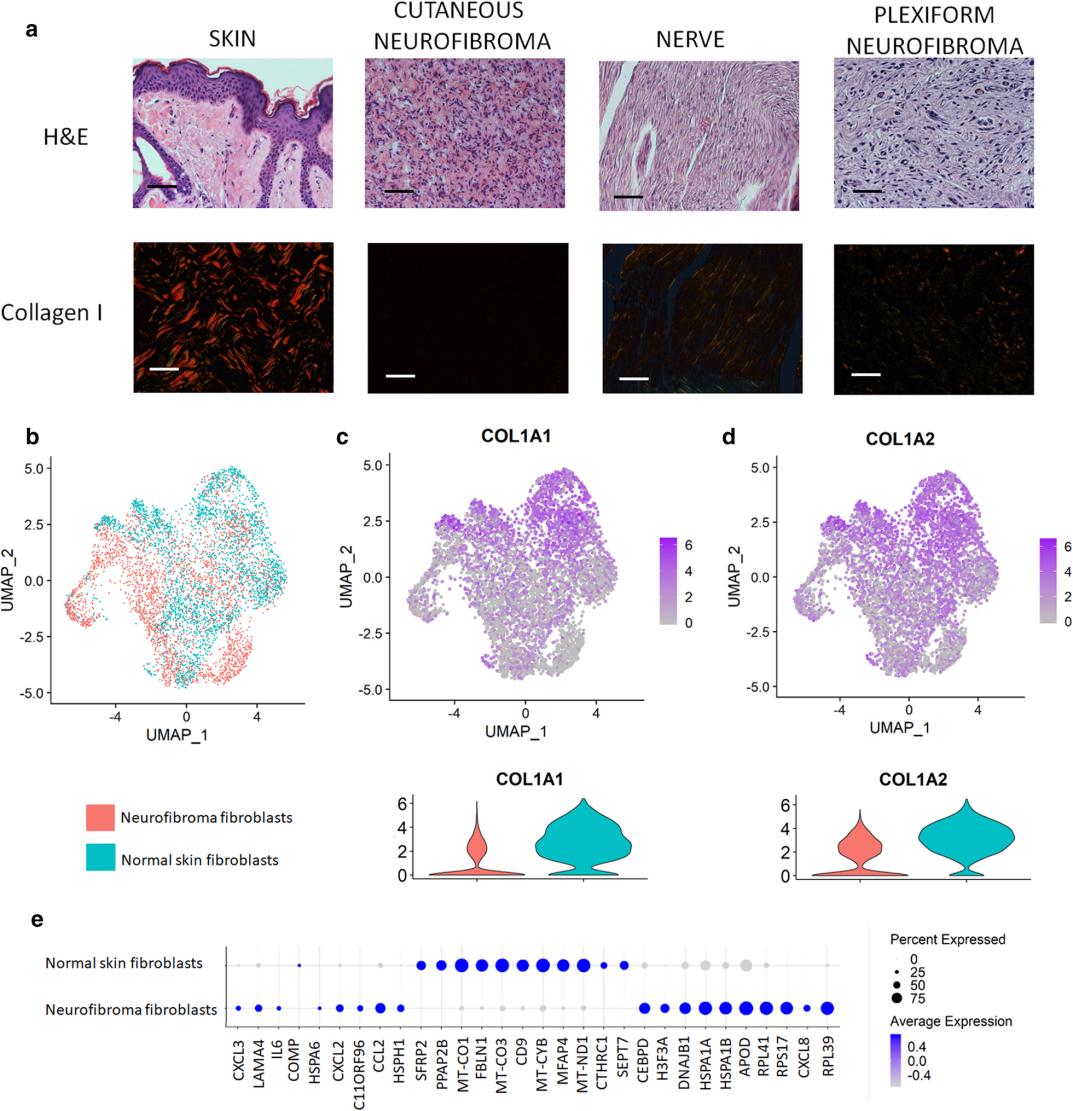
Neurofibroma fibroblasts do not abundantly secrete collagen type I. **a** Expression of collagen type I in human clinical samples [normal skin (skin), cutaneous neurofibroma (cNF), normal peripheral nerve (nerve) and plexiform neurofibroma (pNF)] by Sirius Red staining. **b** Feature plots of the single cell dataset from normal skin fibroblasts (30) (blue) merged with our human neurofibroma fibroblasts (pink). **c**, **d** Feature plots from B (upper) and corresponding violin plot (bottom) of **c** *COL1A1* and **d** *COL1A2*. The intensity of the purple color indicates the normalized level of gene expression. **e** Dot plot representing the differentially expressed genes between neurofibroma fibroblasts and normal skin fibroblasts. Bar = 50 um

Surprisingly, we observed that both genes encoding type I collagen (*COL1A1* and *COL1A2*) were not significantly overexpressed in cNF compared to normal skin margin (Fig. 3c). This prompted us to further investigate the expression of type I collagen using two additional and independent approaches. First, we stained normal skin (i.e. from non-NF1 patients) as well as cNF, normal nerve, and pNF with Sirius Red. When polarized filters are used, collagen type I can be detected specifically as a bright red signal [29]. Sirius Red staining showed abundant deposition of collagen type I in normal skin, whereas this type of collagen is virtually absent from cNF and pNF (Fig. 4a). Second, we took advantage of a published normal skin scRNAseq dataset produced by the same technology [30] to confirm the trend we observed by qPCR (Fig. 3c) and Sirius Red staining (Fig. 4a). To do so, we extracted the transcriptomic data associated with normal skin fibroblasts [30] and integrated them with the transcriptomic data associated with our fibroblast cluster found in Fig. 1a using Seurat. Intriguingly, normal and neurofibroma fibroblasts do not cluster separately and have a very similar gene expression profile (Fig. 4b, e). Collagen type I is expressed by both normal skin fibroblasts and neurofibroma fibroblasts and there is a trend toward lower expression in neurofibroma fibroblasts although it is not statistically significant after false discovery adjustment (Fig. 4c, d). Thus, neurofibroma fibroblasts do not abundantly secrete collagen type I.

### A sub population of neurofibroma fibroblasts secrete collagen type VI

**Fig. 5:**
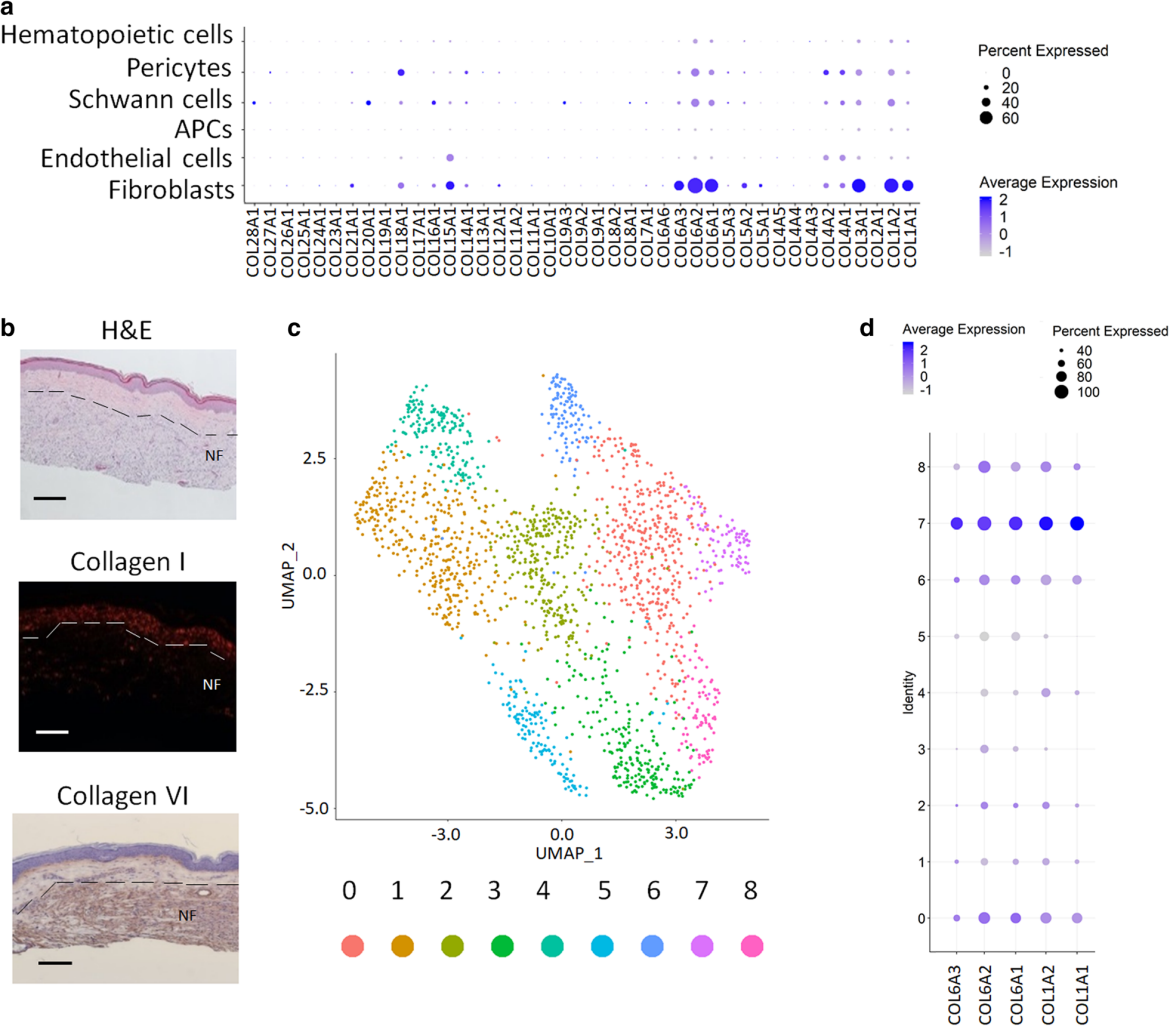
A sub population of neurofibroma fibroblasts secrete collagen type VI. **a** Dot plot representing the shared and common collagen genes expressed in neurofibroma hematopoietic cells, pericytes, Schwann cells, antigen-presenting cells (APCs), endothelial cells, and fibroblasts. Undetectable genes in all six clusters are omitted. **b** Histological characterization of cutaneous neurofibroma by H&E staining (upper), collagen I staining using Sirius Red (middle), and immunohistochemistry using anti-collagen VI antibody (bottom). **c** Subclustering analysis of the neurofibroma fibroblast cluster as found in Fig. 1a shows nine subclusters. **d** Dot plots of collagen type I (COL1A1, COL1A2) and collagen type VI (COL6A1, COL6A2, COL6A3) genes defining the distribution of expression in neurofibroma fibroblasts. The intensity of the purple color indicates the normalized level of gene expression

If it is not collagen I, then what collagen is the predominant type in neurofibroma? To answer this question, we systematically analyzed all collagen genes across the six cell types found in neurofibroma tumor microenvironment. We discovered that the three collagen VI genes (*COL6A1*, *COL6A2*, *COL6A3*) are abundantly expressed in neurofibroma fibroblasts (Fig. 5a). The presence of collagen VI was validated by immunohistochemistry, strongly suggesting that collagen VI is an important component of neurofibroma (Fig. 5b). To evaluate if distinct populations of fibroblasts within the fibroblast cluster express collagen I or VI or a sub-population co-express both, we performed a subclustering analysis exclusively on the fibroblast cluster (Fig. 5c, Additional file 1: Figure S3). Looking at the expression level of COL1A1, COL1A2, COL6A1, COL6A2, COL6A3, the results indicate that a sub population of neurofibroma fibroblasts express both collagen type I and VI whereas a low fraction of the neurofibroma fibroblasts sub-population solely express collagen type I or collagen type VI (Fig. 5d, Additional file 1: Figure S3).

## Discussion

Here, we found 115 matrisome genes expressed in cNF. Importantly, all six neurofibroma cell types (Schwann cells, fibroblasts, endothelial cells, pericytes, APCs, and cells of hematopoietic origin) contribute to the neurofibroma matrisome.

Fibroblasts are defined as extracellular matrix producers, and the presence of collagen is indisputable in neurofibroma. With now 48 genes encoding collagen type I to XXVIII [31], the exact type deposited in neurofibroma microenvironment was unclear. Using a transcriptomic approach, we discovered that collagen VI is among the most highly expressed collagen type in neurofibroma. Collagen VI is usually found at the basement membrane, a specialized area of many polarized cells such as the epidermis-dermis border in the skin; pericytes, and endothelial cells in vasculature; and the Schwann cells wrapping neuronal axons in peripheral nerves. Due to its unique supramolecular assembly, collagen VI helps to maintain basement membrane integrity. In the skin, it is mainly produced by fibroblasts, but in peripheral nerve, it is secreted by Schwann cells. Collagen VI promotes inflammation and angiogenesis, and collagen VI null mice display less tumorigenesis than their wild type littermates [9, 32], whereas collagen VI overexpression enhances it [33]. Here, collagen VI is mainly expressed by a sub-population of neurofibroma fibroblasts. The exact paracrine signaling and receptor involved are currently under investigation. Altogether, it indicates that collagen VI is produced by neurofibroma fibroblasts and may function as a pro-tumorigenic signal via an unknown mechanism.

Once solid tumors reach a certain size, angiogenesis is key for continued growth, and is a hallmark of cancer [34]. Clinically, cNFs are notorious for bleeding when surgically resected [35]. However, while anti-angiogenic treatment is an attractive therapeutic approach in many cancers [36] it was not successful in the context of neurofibroma [2]. The ECM provides critical support for vascular endothelium. Primarily through adhesive interactions with integrins on the endothelial cell surface, ECM provides a scaffold essential for maintaining the organization of endothelial cells into blood vessels [37]. Pericytes wrap around endothelial cells and help maintain vascular homeostasis. As shown in Fig. 2, collagen XVIII expression is predominantly restricted to pericytes among the neurofibroma cell types, and hence is a pericyte neurofibroma marker. Collagen XVIII is found in association with most vascular basement membranes throughout the body [38]. The loss of collagen XVIII from the basement membrane seems to be an early step in tumorigenesis, allowing tumor cells to invade adjacent tissue [39]. We have identified potential endothelial cells markers (e.g. *SERPINE1*, *EGFL7*, *CSF3*) based on their low expression in other non-endothelial neurofibroma cells (Fig. 2). However, it is unclear if those markers would discriminate between normal and neurofibroma vessels. By analogy to neurofibroma fibroblasts, the absence of neurofibroma endothelial cell markers has slowed the investigation of neurofibroma vascular biology and this area of study has remained largely unexplored [40, 41].

As shown in Fig. 2, CCL5 is specifically expressed by CD45-positive hematopoietic cells. In general, CCL5 functions as a chemo-attractant for a variety of leukocytes (e.g. T cells, macrophages) into an inflammatory site through the receptors CCR5 and CCR1. In mouse neurofibroma, CCL5 is mainly expressed by macrophages and Schwann cells [42]. In mouse optic glioma, another NF1 benign tumor, CCL5 is expressed in microglia (the central nervous system equivalent of macrophages) [43]. CCL5 appears to be critical for NF1-related tumors, but the exact mechanism and paracrine signaling is not yet clear [2]. Unfortunately, the relatively small number of CD45-positive cells made it impossible to distinguish the subtype of immune cell (e.g. macrophage, mast cells, T cells) expressing CCL5 in our dataset.

## Conclusion

In summary, we performed systematic profiling of the cNF matrisome. It revealed that all cell types contribute to the matrisome and identified potential markers for cell types within the cNF microenvironment. We also discovered that classic pro-fibrogenic myofibroblasts secreting collagen type I are rare in neurofibroma. In contrast, collagen VI is a pro-tumorigenic ECM mainly secreted by neurofibroma fibroblasts. This work provides insights into the cNF matrisome and offers a molecular foothold to further explore the biology of the cNF microenvironment.

## Methods

### qPCR

Gene expression was determined as previously described [44]. Briefly, RNA extraction was performed using the TRIzol reagent, reverse transcription was performed using iScript Select cDNA synthesis kit, and qPCR was performed on a Bio-Rad FX96 apparatus using Bio-Rad iSCRIPT master mix and the following qPCR primers: *COL1A1* (fwd: GCGAGAGCATGACCGATGGA, rev: GGTCAGCTGGATGGCCACAT); *COL1A2* (fwd: CTGGTCGTGATGGCAACCCT, rev: TAACCGCGCTCTCCCTTGTG); *COL3A1* ( fwd: AATGGTGCTCCTGGACTGCG, rev: ATACCAGCCTCACCGCGTTC); *CTGF* (fwd: ACCTGTGCCTGCCATTACAA, rev: GCTTCATGCCATGTCTCCGT); *FN1*-EDA (fwd: ACTATTGAAGGCTTGCAGCCCA, rev: TGCAGCTCTGCAGTGTCTTCTT); *LOX* (fwd: CTTGCACGTTTCCAATCGCA, rev: GTTACACAAGCCGTTCTGGC); *LOXL2* (fwd: CCAGTGTGGTCTGCAGAGAG, rev: CTCGTTGAGGTGGATGGGTC) and normalized with *GAPDH* (fwd: AGGGCTGCTTTTAACTCTGGT, rev: CCCCACTTGATTTT GGAGGGA).

### Histological characterization

Tissues were processed as described in [44]. Briefly, tissues were fixed in 10% formalin, embedded in paraffin, sectioned at 5 um, and mounted on glass slides.

Histochemical staining was performed as described in [44]. Briefly, tissue slides were deparaffinized, progressively rehydrated, and stained with hematoxylin (2 min) followed by high definition (10 s), bluing agent (10 s), and eosin (H&E staining) or a solution of Sirius Red (0.5 g of Direct Red80 dissolved in 500 mL of saturated picric acid) for 1 h (collagen staining). Finally, tissue slides were progressively dehydrated and coverslipped.

Immunohistochemistry was performed as described in [44]. Briefly, tissue slides were blocked, incubated with primary antibodies [rabbit anti-SMA (Novus, NB600-531); rabbit anti-COL11A1 (ThermoScientific; PA5-68410); rabbit anti-FAP (Abcam; ab53066); rabbit anti-collagen VI (Abcam; ab6588)] diluted in 3% donkey serum (16 h, 4 °C), rinsed in PBS, incubated with secondary antibodies coupled to biotin and diluted in 3% donkey serum (1 h). They were then rinsed again in PBS, incubated with a premixture of avidin and biotin (following Vecta Stain Elite ABC kit procedure) rinsed again in PBS, and visualized by adding the DAB substrate (following Vecta Stain Elite ABC kit procedure). Finally, reactions were quenched in distilled water, and tissue slides were counterstained with hematoxylin, dehydrated, and coverslipped. A brown precipitate was deposited on positive cells.

### Single-cell RNA sequencing

Based on the whole skin dissociation kit (Miltenyi Biotec, Cat. No. 130-101-540), human skin was harvested and immediately immersed in ice-cold DMEM (Gibco, 12634-010). Next, a 4 mm × 4 mm skin piece was placed into ice cold buffer L (435 uL) containing freshly added enzyme P (2.5 uL), enzyme D (10 uL) and enzyme A (0.5 uL). Next, the tube was incubated for 22 h at 37 °C and quenched with ice cold culture medium (500 uL). Next, the digested tissue was minced into 1 mm pieces, incubated 15 min on ice and shaken vigorously every 5 min. After 15 min, the tube was spun (2000 rpm, 5 min, 4 °C), the supernatant discarded, and the pellet resuspended in ice cold complete culture medium (1 mL). The cell suspension was filtered through a 40 um cell strainer, washed with ice cold complete medium (4 mL), and spun at 2000 rpm for 5 min at 4 °C. The supernatant was discarded and the pellet washed again using ice cold 0.04% BSA in PBS (1 mL). Finally, cell count and viability were assessed by hematocytometer (trypan blue). A freshly prepared single cell suspension of ~ 10,000 cells per sample was loaded into a 10X Genomics Chromium controller for transcript barcoding and sequenced on an Illumina HiSeq sequencer. The expression data has been deposited in NCBI's Gene Expression Omnibus (GEO) and is accessible through GEP series accession number GSE163028.

Cell Ranger version 3.0.0 (10× Genomics) was used to process the raw sequencing data. Briefly, raw BCL files were converted to FASTQ files and aligned to the human Grch38 reference transcriptome. Transcript counts of each cell were quantified using barcoded UMI and 10xBC sequences. The gene x cell expression matrices were loaded to the R package Seurat version 3.0.0 for downstream analyses. Cells with low quality were filtered out based on at least 200 genes being detected per 1000 UMIs and mitochondrial gene content. Only those genes found in more than three cells were retained. "LogNormalize" Seurat default global-scaling normalization method was performed. With the above filters in place, we obtained 19,734 genes from 17,132 cells from the three neurofibroma samples combined and 15,607 genes from 2563 cells from the normal skin samples [30] combined. The highly variable features (genes) for this data were then calculated with “FindVariableFeatures" in Seurat, which uses a mean variability plot. The average expression and dispersion per feature are calculated, and features are divided into bins to get z-scores for dispersion per bin. After regressing out the number of UMI and percentage of mitochondrial gene content, the resultant data was scaled, and the dimensional reduction was performed with principal component analysis and visualization using UMAP plots. The QC results can be found in the Additional file 1: Figures S4, S5 and S6. The number of principal components (n = 10) to use in the downstream analysis was calculated based on a Jackstraw and elbow plot of the same. For each sample, a Shared Nearest Neighbor (SNN) Graph was constructed with “FindNeighbors” in Seurat by determining the k-nearest neighbors of each cell. The clusters were then identified by optimizing this SNN modularity using the “FindClusters" function. This allowed for sensitive detection of rare cell types. We obtained five clusters for each sample with a resolution of 0.3. Due to the variability in the cell numbers obtained from the samples, we randomly subsampled 1000 cells per sample to avoid sample cell number bias in visualization and explored the UMAP figures for the cell types. We verified the same for the original dataset (Additional file 1: Figure S1). The differential expression for any of the six clusters over the remaining five was carried out using Wilcoxon Rank Sum test in Seurat [45]. The genes identified as relatively overexpressed in a cluster as compared to all other cells in a sample were termed “markers”. Taking these markers and their functional categories into consideration, these six clusters were identified as six cell types (Fibroblasts, Endothelial cells, Schwann cells, Pericytes, APCs, and Hematopoietic cells). The conserved markers per cell type were also identified using the FindConservedMarkers function from Seurat, which shows genes that are consistently overexpressed in a cell type compared to other cell types across all three samples. To analyze the clusters and cell types in all the tumor samples, they were combined using the method described by Stuart et al. [46]. Canonical correlation analysis was applied to identify correspondences between samples and create a standard reference. To carry out further studies in fibroblasts, we selected only the fibroblast cells from the combined tumor data, randomly subsampled to match the total number of cells from the normal skin sample and compared them against the normal skin sample using the above method [46].

### Human tissues

Human subjects and all sample collection (normal skin, cNF at globular stage and normal margin, keloids) and use were approved by the Institutional Review Board at The University of Texas Southwestern Medical Center and conformed to NIH guidelines. Written informed consent was obtained from patients. Normal peripheral nerve paraffin block was purchased from US BioMax.

## Supplementary information

**Additional file 1.** Supplementary Figures S1–S6.

**Additional file 2.** Supplementary Table S1.
