# Supplementary material for: Human cutaneous neurofibroma matrisome revealed by single-cell RNA sequencing
Source: Acta Neuropathol Commun. 2021 Jan 7;9:11. doi: 10.1186/s40478-020-01103-4 (PMC7792184; doi:10.1186/s40478-020-01103-4)
Supplement: Supplementary file 1 — Additional file 1. Supplementary Figures S1–S6. [file 40478_2020_1103_MOESM1_ESM.pdf]

Figure S1. Single cell analysis quality metrics related to figure 1

A

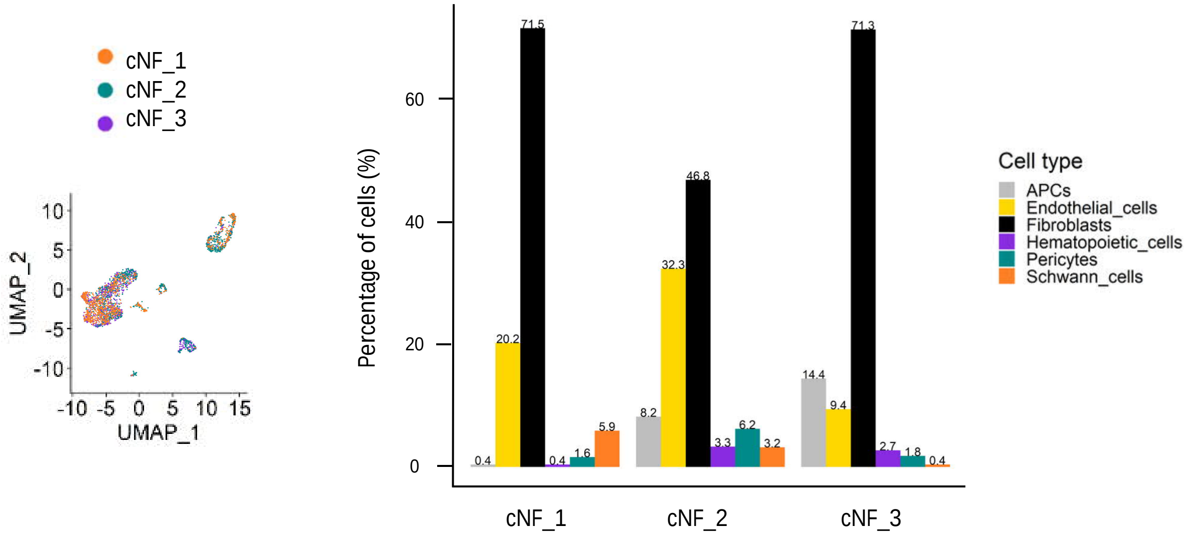

B

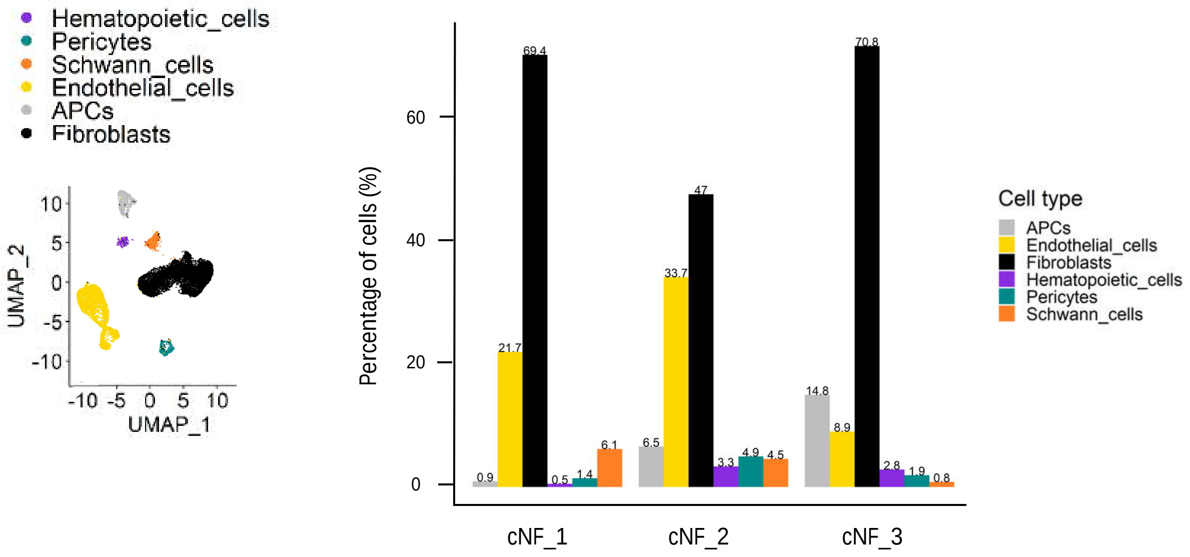

Supplementary Figure 1. Single cell analysis quality metrics related to figure 1.

A Uniform Manifold Approximation and Projection (UMAP) shows groupings of three human cutaneous neurofibroma cell populations totaling 3,000 cells (random sampling of 1000 cells per sample). Each point represents a cell. Cells are color-coded according to the sample identity (left panel). Percentage of cells per cell type and sample (right panel). **B.** Uniform Manifold Approximation and Projection (UMAP) shows groupings of three human cutaneous neurofibroma cell populations totaling 17,312 cells. Each point represents a cell. Cells are color-coded according to the sample identity (left panel). Percentage of cells per cell type and sample (right panel).

Figure S2. Alternative cell-type specific markers for neurofibroma

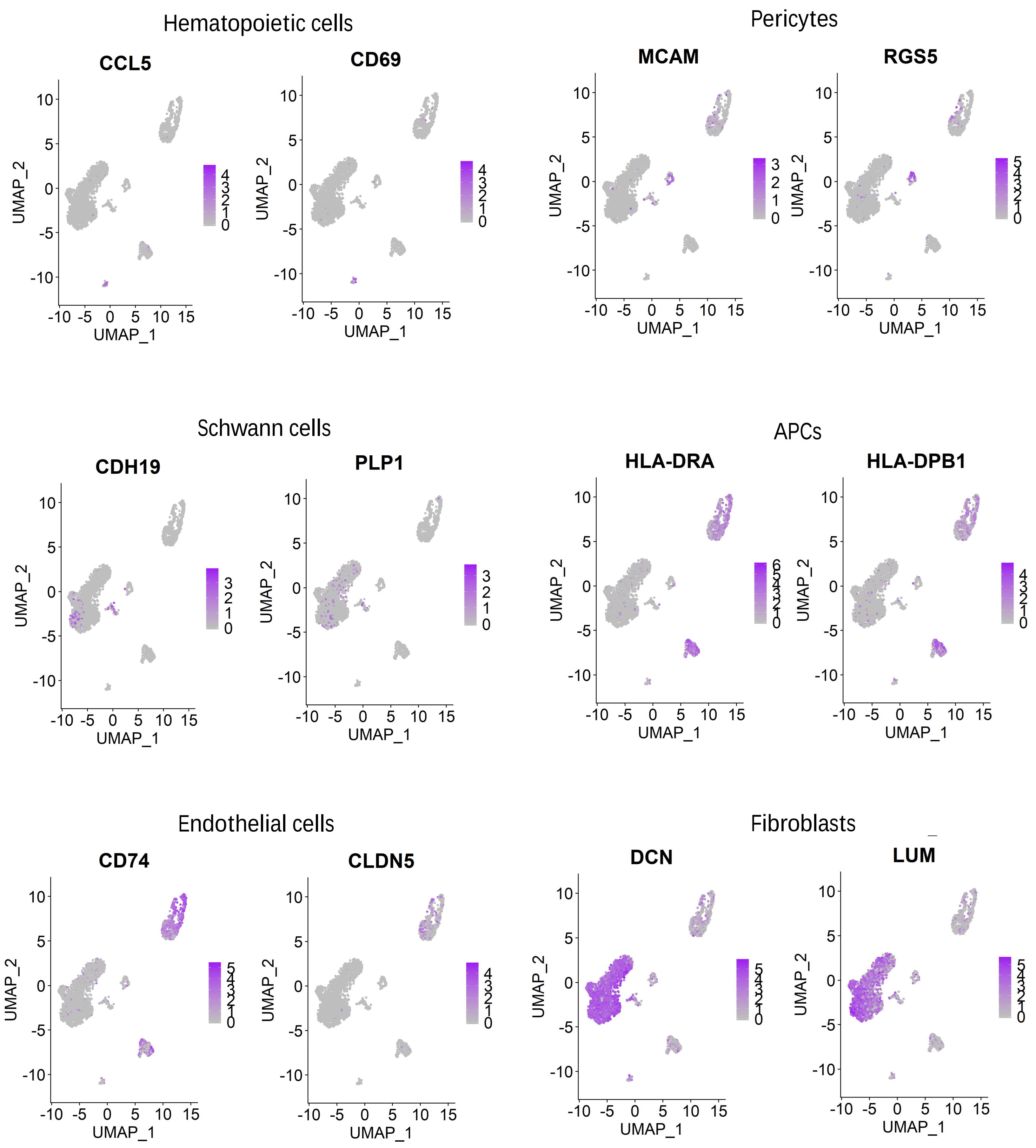

Supplementary Figure 2. Alternative cell-type specific markers of neurofibroma cells. Feature plots of genes defining different cell types in human cutaneous neurofibroma. The intensity of the purple color indicates the normalized level of gene expression.

Figure S3. Subclustering analysis of neurofibroma fibroblasts

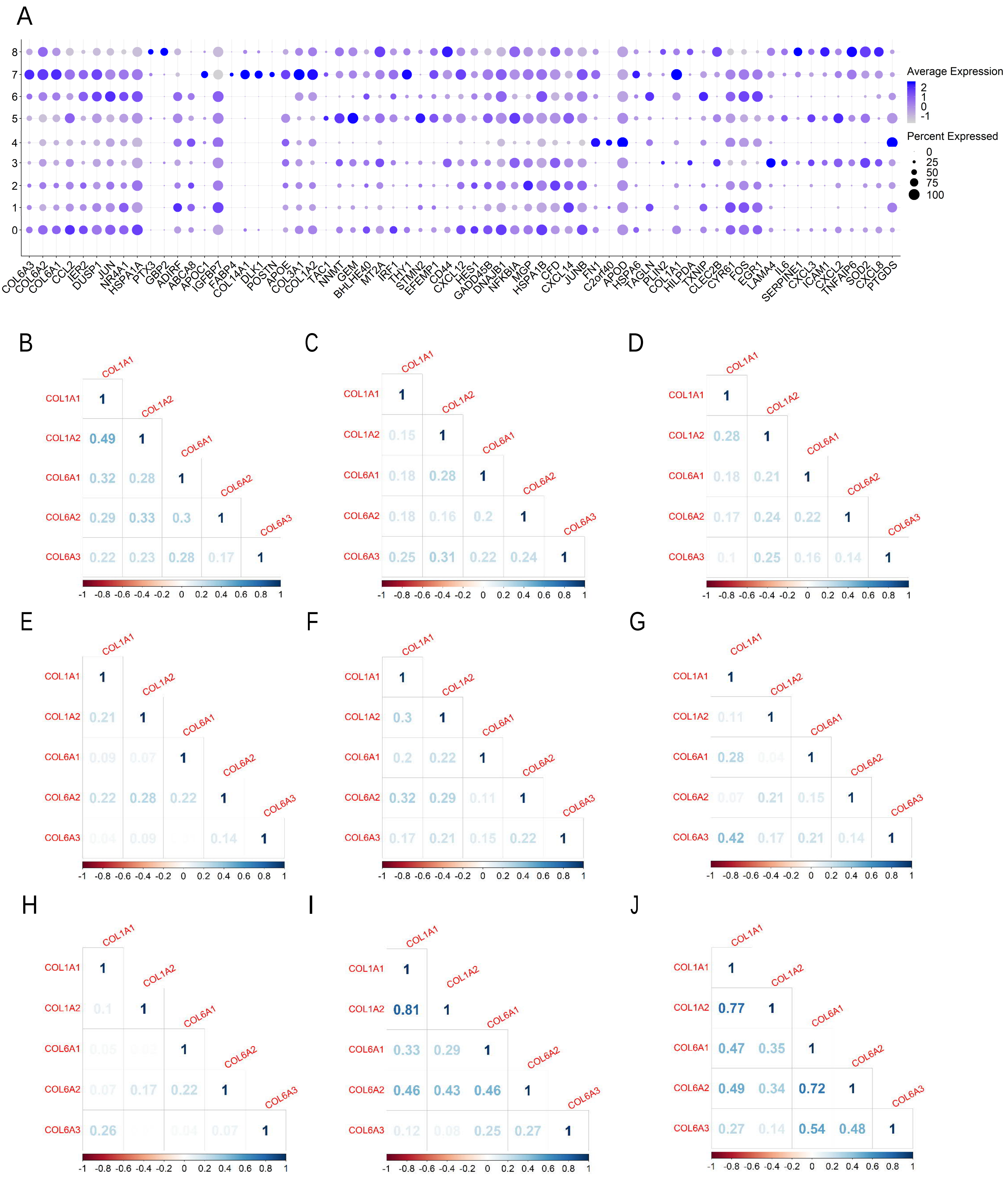

Supplementary Figure 3. Subclustering analysis of neurofibroma fibroblasts. A. Dot plot representing the differentially expressed genes within the neurofibroma fibroblast cluster. **B-J** Correlation plots. **B** Subcluster 0. **C** Subcluster 1**. D** Subcluster 2. **E** Subcluster 3. **F** Subcluster 4**. G** Subcluster 5. **H** Subcluster 6. **I** Subcluster 7. **J** Subcluster 8. A value of 1 mean a perfect expression correlation between two genes.

Figure S4. Single cell analysis quality metrics related to the cNF samples

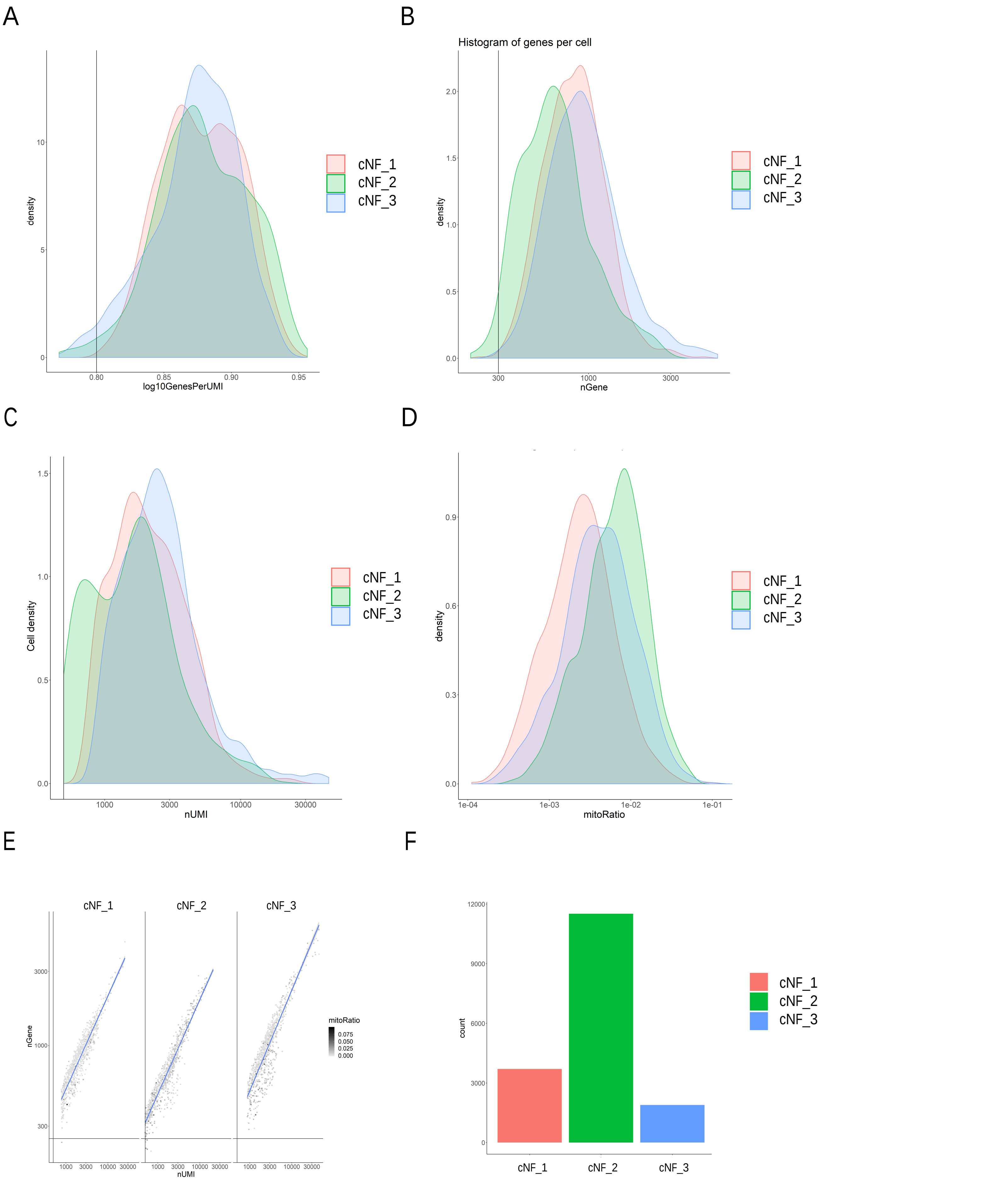

Supplementary Figure 4. Single cell analysis quality metrics related to the cNF samples.

A. Overall complexity. **B.** Histogram of genes per cell. **C.** Number of UMIs/transcripts per cell. **D.** Mitochondrial gene expression per cell. **E.** Correlation between genes and UMIs. **F.** Number of cells per sample.

Figure S5. Single cell analysis quality metrics related to the normal skin samples

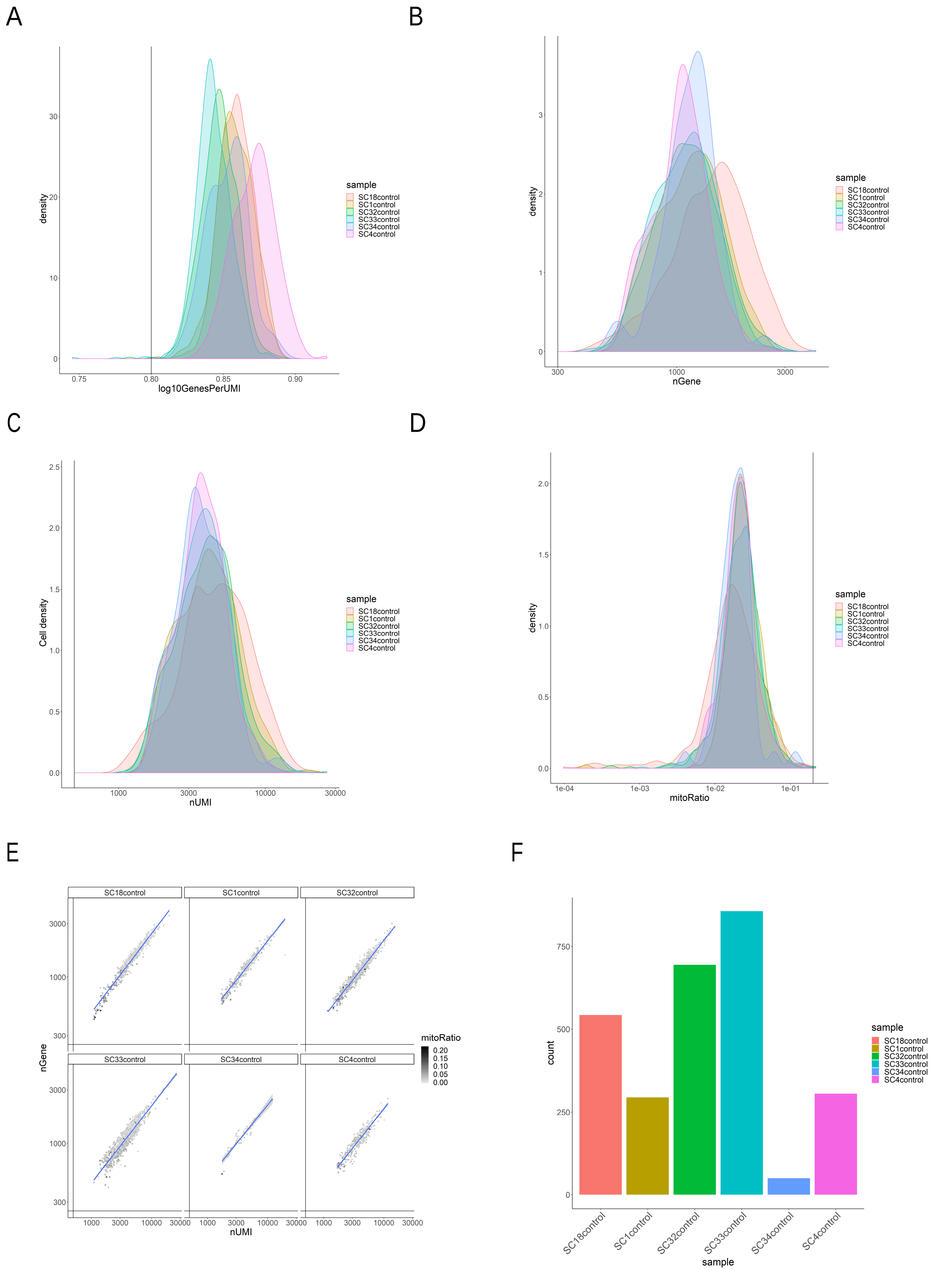

Supplementary Figure 5. Single cell analysis quality metrics related to the normal skin samples.

A. Overall complexity. **B.** Histogram of genes per cell. **C** Number of UMIs/transcripts per cell. **D.** Mitochondrial gene expression per cell. **E.** Correlation between genes and UMIs. **F.** Number of cells per sample.

Figure S6. Single cell analysis quality metrics related to the neurofibroma fibroblast cluster

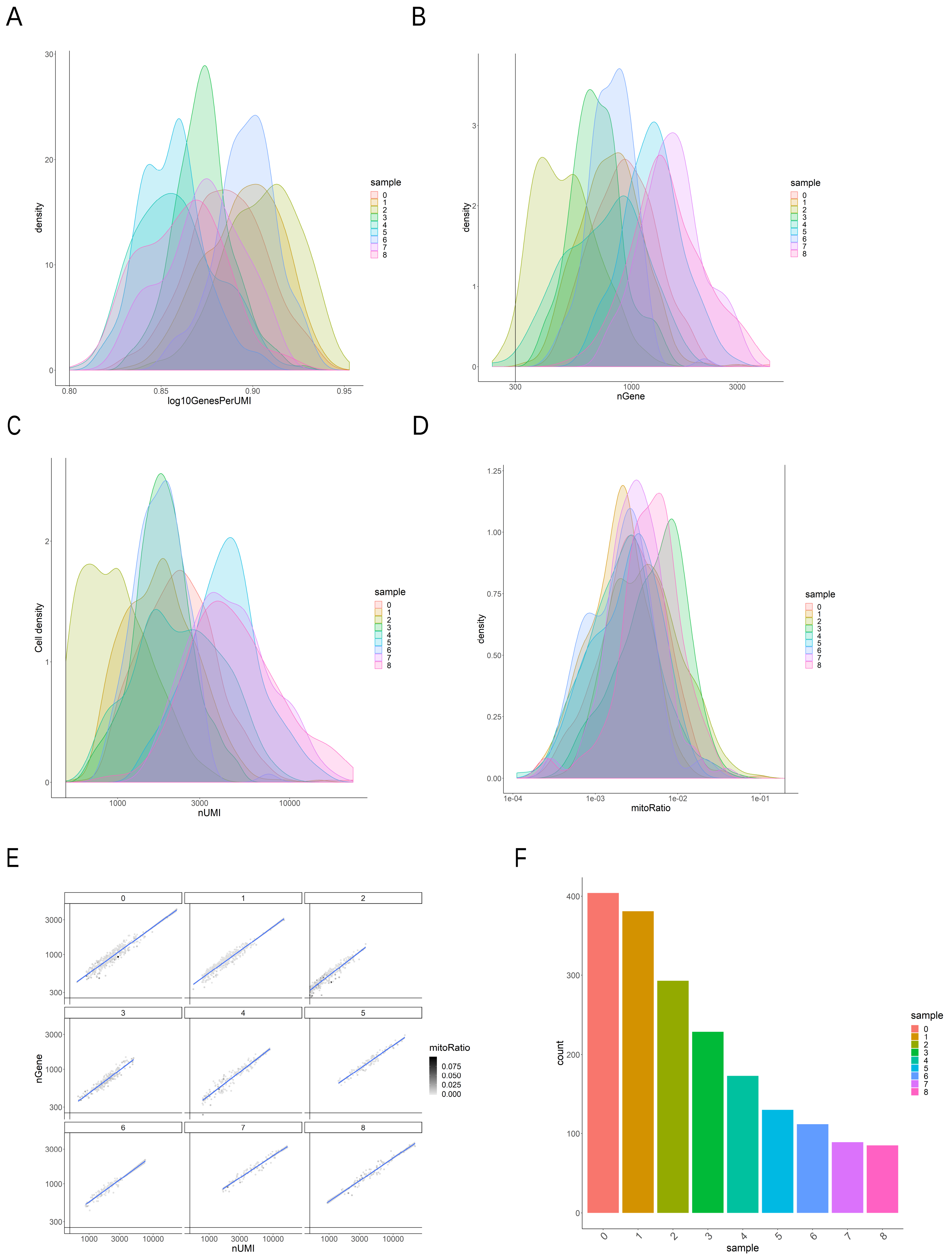

Supplementary Figure 6. Single cell analysis quality metrics related to the neurofibroma fibroblast cluster.

A. Overall complexity. **B.** Histogram of genes per cell. **C** Number of UMIs/transcripts per cell. **D.** Mitochondrial gene expression per cell. **E.** Correlation between genes and UMIs. **F.** Number of cells per sample.
